# Acceptability of Wearable Digital Devices for Remote Continuous Mobility Monitoring in People With Parkinson Disease and Carers: Qualitative Study

**DOI:** 10.2196/98987

**Published:** 2026-10-30

**Authors:** Megan Hanrahan, Jack Lumsdon, Cameron Wilson, Essa Bhadra, Alison Keogh, Tova Gur-Arieh, Alastair Noyce, Alison Yarnall, Lynn Rochester, Ríona Mc Ardle, Katie Brittain

**Affiliations:** 1 Population Health Sciences Institute, Faculty of Medical Sciences, Newcastle University, Newcastle Biomedical Research Building, Clinical Ageing Research Unit (CARU), Campus for Ageing and Vitality, Westgate Road, Newcastle upon Tyne, England, NE4 5PL, United Kingdom, 44 0191 208 7565; 2 NIHR Newcastle Biomedical Research Centre, Newcastle University, Newcastle upon Tyne, England, United Kingdom; 3 Department of Public Health and Primary Care, School of Clinical Medicine, University of Cambridge, Cambridge, England, United Kingdom; 4 Department of Neurology, Barts Health NHS Trust, London, England, United Kingdom; 5 School of Medicine, Trinity College Dublin, Dublin, Leinster, Ireland; 6 Mobilise-D Patient and Public Advisory Group, Newcastle upon Tyne, England, United Kingdom; 7 Preventive Neurology Unit, Wolfson Institute of Preventive Medicine, Queen Mary University of London, London, England, United Kingdom; 8 Translational and Clinical Research Institute, Faculty of Medical Sciences, Newcastle University, Newcastle upon Tyne, England, United Kingdom; 9 Clinical Ageing Research Unit, The Newcastle upon Tyne Hospitals NHS Foundation Trust, Newcastle University, Newcastle upon Tyne, England, United Kingdom

**Keywords:** Parkinson disease, wearable devices, qualitative, carers, digital health technology, acceptability

## Abstract

**Background:**

Parkinson disease (PD) impairs mobility and motor function, making accurate mobility assessment important for effective management and treatment planning. Traditional clinical assessments often rely on periodic clinical evaluations, which may not capture day-to-day symptom fluctuations. Wearable devices offer a promising alternative with continuous, objective tracking of mobility (eg, gait). However, although these tools provide valuable insights for patients and clinicians, their successful implementation depends on their acceptability to users.

**Objective:**

This study aimed to explore the acceptability of wearable devices for remote continuous mobility monitoring in PD from the perspectives of people with PD and informal carers.

**Methods:**

This international qualitative study used purposive sampling to recruit a diverse sample of 72 people with PD and 20 informal carers from the United Kingdom, Ireland, Belgium, Germany, Spain, and the United States. Participants were recruited between June 2023 and February 2026 through multiple community-based routes. One-to-one semistructured interviews, conducted online, via telephone, or in person, explored views on wearable devices for digital mobility assessment. Interview data were analyzed using Braun and Clarke’s 6-phase reflexive thematic analysis.

**Results:**

Most people with PD were generally open to wearable monitoring when it could capture motor fluctuations between “on” and “off” periods that were difficult to convey during clinical appointments, reduce reliance on memory, provide objective evidence to support treatment and medication discussions and self-advocacy, and track changes in mobility over time, which some felt could be reassuring. Acceptability depended on devices having a clear purpose, requiring minimal effort, and being easy to use. Tremor, reduced dexterity, and “off” periods could make fastening straps, navigating menus, and charging devices difficult. Carers valued wearable monitoring for the reassurance and safety it offered, particularly fall detection, and for support with both clinical and everyday decision-making but emphasized that devices must operate passively and integrate into daily caregiving without adding burden.

**Conclusions:**

Wearable mobility monitoring was generally acceptable to people with PD and carers when it offered clear clinical value, fit easily into everyday life, and provided reassurance that remote monitoring would complement rather than replace clinical care. As PD mobility was experienced as a shared concern within the care relationship, the value of monitoring depended on how well it supported the wider care dyad rather than the person with PD alone. These findings suggest that successful implementation will require co-design with people with PD and carers, alongside thoughtful clinical integration, to ensure that wearable technologies are both acceptable and effective in practice.

## Introduction

### Background

Parkinson disease (PD) is a progressive neurodegenerative condition that affects approximately 8.5 million people globally and significantly shapes how people move and participate in everyday life [1]. For many people with PD, maintaining walking ability, balance, and confidence in movement remains a central priority because mobility limitations can reduce independence, participation in daily activities, and quality of life [2-4]. PD is characterized by a range of cardinal motor symptoms, such as bradykinesia, rigidity, and postural instability, which are commonly associated with impaired mobility [5]. In this context, mobility refers to the physical ability to move freely and easily from one place to another [6] and includes other aspects of successful movement, such as balance. As the disease progresses, these difficulties often become more pronounced [7,8]. Mobility may also fluctuate throughout the day, partly relating to medication response and transitions between “on” and “off” states, making movement less predictable and further affecting confidence and participation [2]. Monitoring mobility is therefore valuable for informing condition management, optimizing treatment, and assessing disease progression over time [9-11].

In routine clinical practice, mobility assessment in PD often relies on episodic clinical evaluations and patient self-report [12]. Although standardized rating scales and brief functional assessments are widely used [13], they provide only a snapshot and may fail to capture a true reflection of symptom variability and context-specific challenges [14,15]. People with PD have highlighted this limitation, reporting frustration with assessments that do not reflect their typical functioning [16]. These assessment methods also rely on patients’ memory and are therefore vulnerable to recall bias [16,17]. Consequently, important longitudinal changes in mobility may go undetected, potentially delaying both timely intervention and the diagnosis of PD.

Wearable technologies use inertial sensors, such as accelerometers and gyroscopes, embedded in smartwatches and other body-worn devices to enable continuous, real-world monitoring of movement and gait, which can generate objective and ecologically valid measures of mobility in everyday settings [9,18]. These possibilities have generated considerable enthusiasm for wearable devices as a way to address the limitations of current episodic clinic-based assessment [19,20]. By capturing mobility beyond the clinic, such devices may provide a more accurate picture of everyday functional performance and detect subtle changes that may otherwise go unnoticed between appointments. In PD specifically, wearable devices can record clinically relevant motor information, such as gait variability, tremor, and movement patterns, supporting more precise monitoring of progression and treatment response [9,11,21]. Device configurations vary in sensor type and placement, worn at locations such as the wrist, chest, waist, thigh, shank, feet, head, and lower back; these design choices influence the signals captured and the accuracy of the algorithms used to derive clinically meaningful outcomes [22]. Wearables may also facilitate richer information exchange between clinicians and patients by complementing self-report with objective mobility data, enabling more informed clinical discussions and shared decision-making [23]. Wearable devices are therefore increasingly positioned as promising tools for supporting more responsive, data-informed approaches to PD management.

Successful implementation of wearable devices in PD care depends not only on their technical validity but also on their acceptability to the people who use them. Acceptability is a multifaceted concept encompassing perceptions of burden, comfort, usability, and perceived value and is a key determinant of the uptake and sustained use of health care innovations [24]. In PD, this is especially important because motor and cognitive symptoms may create condition-specific barriers that influence whether and how people are able or willing to engage with wearable technology [25]. For example, tremor or reduced dexterity may make it difficult to fasten straps or manage device setup [26], and concerns about comfort, stigma, privacy, and effort may further reduce willingness to engage, even when potential clinical benefits are recognized [26,27]. These challenges may be especially important for underserved groups, who are often underrepresented in digital health research and may be disproportionately affected by digital exclusion, resulting in disparities in access, motivation, and ability to use digital health technologies [28]. Broader concerns about the lack of diversity in PD research have also been raised more generally, raising questions about whose needs and preferences are reflected in the existing evidence base [29]. Carers must also be considered in this context; in PD, they often play a central role in daily support, symptom management, and clinical discussions [30] and may be directly involved in the use and management of wearable devices. Yet, carers remain much less frequently included as participants in PD digital health research specifically, even where the technologies depend on their involvement.

While a growing body of research has examined the feasibility and technical validity of wearable technology in PD [9,31], comparatively less attention has been paid to how the devices are experienced by people with PD and their carers in everyday life [26,32,33] or whether they are acceptable across groups with differing needs and priorities. Given the variation in device configurations and sensor placements used across PD research, it is also important to understand how such differences may shape perceptions of acceptability in real-world use. Without a clear understanding of acceptability, there is a risk of implementing tools that do not align with users’ needs, capabilities, or preferences, limiting their adoption and impact in practice [24]. More person-centered approaches to wearable technology development and evaluation have been proposed as a route to improving long-term engagement [33], reinforcing the value of exploring user perspectives alongside technical performance. This paper presents qualitative research exploring the perspectives of people with PD and carers on the use of wearable devices for remote continuous mobility monitoring, identifying the barriers and facilitators shaping their use and exploring expectations for how these technologies might be integrated into PD care.

### Aims

The aims of the study were to (1) explore the acceptability of wearable devices for remote continuous mobility monitoring from the perspectives of people with PD and informal carers, (2) identify barriers and facilitators influencing uptake and sustained use of wearable devices in PD care, and (3) examine user preferences and expectations for how wearable devices should be integrated into PD health care.

## Methods

### Study Design

A qualitative approach was taken, using one-to-one semistructured interviews to explore the acceptability of wearable devices for monitoring mobility in PD from the perspectives of those with PD and from those who support or care for someone with PD.

### Setting

This international qualitative study included participants from the United Kingdom, Ireland, Belgium, Germany, Spain, and the United States. Interviews were conducted primarily online via teleconferencing platforms, with additional interviews carried out via telephone and in person.

### Participant Recruitment

Participants were eligible if they self-reported a diagnosis of PD or supported or cared for someone with a diagnosis of PD, were aged ≥18 years, and had the capacity to provide informed consent. Previous experience of wearable devices used for digital health was not a requirement, as these devices are not yet part of routine PD care. Requiring experience would have limited eligibility to former research participants and risked excluding the underserved groups we aimed to reach. Including participants new to wearables helped us to capture the perspectives of those who might first encounter these devices if they were introduced into routine care.

Recruitment took place through multiple routes, including voluntary organizations and charities (eg, Parkinson’s UK, Cure Parkinson’s, and the Michael J Fox Foundation), the VOICE participation platform, social media, online newsletters and forums, and in-person engagement at support groups and exercise classes. Participants were also recruited through the East London Parkinson’s Disease project.

A purposive sampling strategy was used to capture a wide range of lived experiences, prioritizing groups known to be at risk of digital exclusion in health care, as guided by the culture, limiting conditions, education, age, residence, and socioeconomic status (CLEARS) framework [28]: people from rural or socioeconomically deprived areas, those with lower levels of formal education, ethnic minority communities, and those with limiting conditions. The research team also drew on wider evidence and discussion regarding underrepresentation in digital health and PD research to additionally prioritize people with young-onset PD (diagnosed aged ≤50 years), adults aged ≥80 years, and LGBTQIA+ individuals, all of whom are rarely reflected in existing research [34-36]. This approach was taken to ensure a comprehensive and inclusive understanding of acceptability, informed by those typically underrepresented in PD and digital health research.

Purposive sampling was executed through recruitment routes chosen for their reach into specific groups. The research team built relationships with community organizations that already worked with and were trusted by these groups. This approach was informed by 2 patient and public involvement and engagement (PPIE) workshops held with clinicians and researchers external to the project, which helped codevelop recruitment strategies. The composition of the sample was reviewed throughout so that efforts could be focused on groups that remained underrepresented. Participation was further supported by offering interviews online, via telephone, or in person according to participants’ preference and in their preferred languages. Our sample size was guided by the aim to capture a diverse sample, with representation across the targeted characteristics accruing gradually over a 32-month recruitment period.

### Data Collection

Semistructured interviews, lasting on average 45 (SD 16.2; range 19‐109) minutes, explored the acceptability of digital mobility assessments, with a particular focus on wearable devices to monitor mobility in PD. Interviews were conducted in participants’ preferred language; most were in English, while interviews with participants whose preferred language was Bengali, German, or Dutch were conducted by bilingual members of the research team who had received training in the use of the interview guide. Each interview began with a brief demographic questionnaire covering age, gender, ethnicity, postcode, sexuality, employment status, and comorbidities to contextualize responses and support analysis of participant diversity. Postcode was used to derive socioeconomic status through local deprivation indicators (eg, Index of Multiple Deprivation [37], Pobal HP Deprivation Index [38], Area Deprivation Index [39], and Northern Ireland Multiple Deprivation Measure [40]) and to classify rurality (eg, Rural-Urban Classification [41], Am I Rural [42], and Urban Rural Life [43]). Previous experience of wearable devices was explored during the interviews and recorded from the interview data.

The interviews followed semistructured topic guides, which included a dedicated section on wearable device acceptability (Multimedia Appendices 1 and 2). Participants were given a brief verbal explanation of wearable devices used in PD mobility research, drawing on examples from the Mobilise-D project [44,45], including wrist-worn and lower-back sensors. Both were offered as illustrative examples to give participants a sense of what wearable mobility monitoring might involve. Participants were informed that while both device types can monitor mobility, lower-back sensors are generally considered to provide greater measurement accuracy [46,47]. This information was provided so that participants could form an informed view on a device type most would not have encountered, as wrist-worn devices are widely familiar as consumer products, whereas lower-back sensors are used almost exclusively in research. Nonetheless, participants were encouraged to describe what they would hypothetically want from a wearable device, ensuring their responses reflected their own needs and preferences rather than being anchored to any specific device type.

People with PD were asked what they thought about wearing a device to monitor their mobility, with prompts exploring preferred body placement; what they felt would be good about these devices, including what they would want the device to capture; any concerns, with prompts covering comfort, ease of use, and data privacy; and whether they would be willing to wear the device and, if so, for how long. Carers were asked whether they currently used technology to support their care partner; what they thought about using a device to monitor the mobility of the person they supported; how these devices would be helpful, including any specific features that would be useful; any concerns, with prompts covering comfort, ease of use, and privacy; and how the devices might support existing caring responsibilities and routines.

### Patient and Public Involvement and Engagement

PPIE was embedded throughout the project, and reporting aligns with minimum PPIE recommendations [48]. A public contributor living with PD was a coapplicant on the funding application and contributed to the development of the interview topic guide. During recruitment, we held 2 PPIE workshops with clinicians and researchers external to the project to co-develop recruitment strategies, with particular attention to supporting participation from underserved groups. Pilot interviews were conducted with 2 public contributors, one person with PD and one informal carer, to ensure the language, structure, and content of the guides were clear and relevant. Their feedback highlighted areas where wording could be simplified, suggested additional questions and prompts to elicit deeper responses, and identified opportunities to improve question ordering. These insights directly informed iterative refinements to the language and flow of the topic guides. To assess feasibility for participants whose preferred language was not English, we piloted 1 interview using interpreter support. However, this approach disrupted the natural flow of conversation, making it difficult to maintain dialogue and follow up on responses in the moment, which is important for data quality in qualitative research. We therefore trained a small number of researchers to conduct interviews in participants’ preferred languages using the same topic guide, thereby broadening accessibility while maintaining consistency across interviews. One public contributor was also invited to comment on a draft of the final paper. These activities reflected a collaborative rather than consultative approach, involving public contributors across multiple stages of the study.

### Data Analysis

All interviews were analyzed using reflexive thematic analysis (RTA) to develop an in-depth understanding of the acceptability of digital mobility assessments, specifically wearable devices, from the perspectives of people with PD and carers. This approach allows for an interpretative account of acceptability grounded in participants’ own priorities. Analysis followed Braun and Clarke’s 6-phase process [49], which involves familiarizing oneself with the data, generating initial codes, searching for themes, reviewing themes, defining and naming themes, and producing the report.

Transcripts were read repeatedly to support familiarization with the data, which was further supported by the lead analyst (MH) having conducted 43 of the 92 interviews. Analysis ran alongside a 32-month period of data collection (June 2023 to February 2026). Early coding was undertaken collaboratively by MH, JL, and KB, who jointly coded a small subset of transcripts in shared sessions to build familiarity with the data and to explore initial codes and interpretations. Codes were generated and discussed as the transcripts were worked through together. MH then took the analysis forward across the full dataset, developing and revising codes inductively using NVivo (version 15; Lumivero) and Microsoft Word. Initial impressions were documented after each interview, and accounts that aligned with or diverged from emerging patterns were noted; this was revisited alongside the transcripts during analysis. Theme development and interpretation were discussed in regular sessions with RMA, KB, and JL. Consistent with RTA, collaboration was used to enrich the lead analyst’s developing interpretations rather than to establish coding consensus. Rigor and trustworthiness were supported through sustained engagement with the data, documentation of developing interpretations, ongoing reflexivity, and team discussion. Interview data from people with PD and carers were analyzed separately to develop within-group themes before exploring perspectives across groups to identify similarities and differences. In developing themes, we distinguished accounts grounded in previous use of wearable technology from those anticipating use; both informed the analysis, and where an account drew on previous experience, this is indicated in the *Results* section. Participant quotations are also presented with a participant identifier, gender, age, and length of diagnosis or diagnosis of person cared for.

### Reflexivity

MH specializes in digital health and PD research, and the wider team brings complementary expertise spanning public health, neurology, clinical medicine, rehabilitation, psychology, aging, and social science. This collective expertise supported informed data collection and probing during interviews, although it may also have shaped how wearable technology is viewed in PD care. To address this, we took reflexive steps such as documenting notes while coding and developing themes, discussing theme development and interpretations with the wider team to enhance reflexivity, and noting down and revisiting assumptions.

### Ethical Considerations

Ethical approval was granted by Newcastle University’s Faculty of Medical Sciences Research Ethics Committee (references 2562_2/31981/2024 and 48672/2025) and Southwest—Central Bristol Research Ethics Committee (references: 39/SW/0255; IRAS ID 242395). All participants provided informed consent before taking part, including consent for audio recording, which was further reconfirmed on recording at the beginning of the interview. Online interviews were recorded using Zoom (Zoom Communications, Inc) and Microsoft Teams; telephone and in-person interviews were recorded, and recordings were transferred to secure Newcastle University storage and then deleted. Audio files and transcripts were stored on a university shared drive with restricted access. Transcription was completed by either UK Transcription under a confidentiality agreement or the researchers who had conducted the interview. Transcripts were checked for accuracy and anonymized by removing names and any other potentially identifying information before analysis. Participants were reminded that their involvement was voluntary and that they could stop the interview at any time without giving a reason. No financial incentives were provided.

## Results

In total, interviews were conducted with 72 people with PD and 20 informal carers. Previous experience of a wearable device was reported by 61% (44/72) of people with PD and 50% (10/20) of carers. An overview of participant characteristics is provided in Table 1.

**Table 1.** Participant demographics for people with Parkinson disease (PD) and carers.

Demographics	People with PD (n=72)	Carers (n=20)
Age (years), median (minimum to maximum)	64.5 (42-81)	67.5 (30-88)
≤50, n (%)	9 (13)	1 (5)
≥80, n (%)	2 (3)	2 (10)
Length of diagnosis for self or for person cared for (years), mean (SD, minimum to maximum)	5.8 (3.9, 0.5‐20)	10.2 (6.6, 1.5‐20)
Gender, n (%)		
Women	31 (43)	15 (75)
Men	41 (57)	5 (25)
Ethnicity, n (%)		
White	62 (86)	19 (95)
South Asian	9 (13)	—^a^
Arab	1 (1)	—
Ashkenazi Jewish	—	1 (5)
Country of residence, n (%)		
UK	57 (79)	20 (100)
United States	7 (10)	—
Belgium	4 (6)	—
Germany	2 (3)	—
Ireland	1 (1)	—
Spain	1 (1)	—
Rurality, n (%)		
Urban	50 (69)	16 (80)
Rural	22 (31)	4 (20)
Socioeconomic status area, n (%)		
Less deprived	31 (43)	13 (65)
Moderately deprived	27 (38)	5 (25)
More deprived	14 (19)	2 (10)
Education, n (%)		
Compulsory education (eg, GCSE/O-level^b^, equivalent)	6 (8)	2 (11)
Further education (eg, A-levels, BTEC^c^, equivalent)	4 (6)	3 (15)
Higher education below degree (eg, HNC^d^, HND^e^, diploma)	16 (22)	1 (5)
Undergraduate degree	15 (21)	10 (50)
Postgraduate degree	25 (35)	4 (21)
Not reported	6 (8)	—
Employment status, n (%)		
Retired	50 (69)	14 (70)
Paid work	11 (15)	5 (25)
Not working, unemployed, or sick leave	5 (7)	1 (5)
Not reported	6 (8)	—
Sexual orientation, n (%)		
Heterosexual or straight	62 (86)	20 (100)
Gay	10 (14)	—
Prior wearable device experience, n (%)		
PD-related device (research or clinical)	28 (39)	4 (20)
Consumer device only (eg, smartwatch or fitness tracker)	16 (22)	6 (30)
No prior use	28 (39)	10 (50)

a Not applicable.

b GCSE/O-level: General Certificate of Secondary Education/General Certificate of Education Ordinary Level.

c BTEC: Business and Technology Education Council.

d HNC: Higher National Certificate.

e HND: Higher National Diploma.

Three core themes and associated subthemes were developed that captured how wearable devices were perceived and what shaped their acceptability across both people with PD and carers. Two themes, wearable data as a window into the lived reality of PD and making technology fit the person, were relevant to both people with PD and carers and are therefore presented with integrated viewpoints. The third theme, reassurance, responsibility, and the burden of care, was developed through the analysis of carer accounts and captured experiences specific to carers.

All 3 themes were evident across the sample, including among participants with the characteristics prioritized in recruitment. As the sample was designed to capture diverse perspectives rather than to compare subgroups, we do not interpret this consistency as meaning participants’ experiences were the same; instead, it suggests that the key aspects of acceptability were shared, even where individual circumstances differed. Themes, subthemes, and associated concepts are illustrated in Table 2.

**Table 2.** Themes, subthemes, and associated concepts relating to the acceptability of wearable devices for remote continuous mobility monitoring in Parkinson disease (PD).

Theme and subtheme	Concepts
Wearable data as a window into the lived reality of PD	
Beyond the clinical snapshot	Clinical assessments viewed as unrepresentative of everyday function, continuous data valued for capturing real-world symptom fluctuations and reducing reliance on recall
Data as personal evidence	Data used for self-advocacy and informing treatment decisions, expectations of data ownership and a desire for interpretable outputs
Complementing, not replacing, care	Potential for better-informed consultations, concerns that monitoring could replace current care
Making technology fit the person	
Usability defined by the difficult moments	Device use during “off” periods, preference for passive monitoring and accommodating individual health needs
Managing visibility and discretion	Device appearance in public settings
The emotional weight of monitoring	Data as reassuring or as a source of distress
Reassurance, responsibility, and the burden of care	
Relief from constant vigilance	Fall detection and alerting valued as a safety net and objective awareness of difficulties
Supporting care without adding to it	False alarms and alert fatigue, device management falling to carers, preference for passive monitoring, recognition of carers as stakeholders, feasibility across formal care settings

### Wearable Data as a Window Into the Lived Reality of PD

#### Overview

Across both groups, wearable monitoring was valued for its potential to address the perceived limitations of episodic clinical assessment. Participants described clinic-based assessments as providing an unrepresentative snapshot of functioning, whereas continuous monitoring was expected to generate data they considered more objective and reflective of mobility in everyday life. This perceived value was based on capturing symptom fluctuations, supporting agency through access to interpretable data, and enabling more productive clinical appointments.

#### Beyond the Clinical Snapshot

For people with PD, the perceived value of continuous monitoring began with a critique of current episodic clinical assessment. Several described clinical appointments as *“*artificial” contexts in which they appeared better than they typically were, either because they were being observed or because they consciously tried to demonstrate particular symptoms to the clinician. One participant explained how this meant the clinician would not see how his symptoms usually presented in everyday life:

*...you perform for [the clinician]. You’re going, like, “Look at this—all working,” and they go, “Great.” Whereas it’s not real...he doesn’t see the real stuff that goes on. He just sees the performance you put on for them...So, the wearable thing, if it’s uploading data to them, happy days, then they can see how you are in the world.*

[P40, male, 49 years, 5 years diagnosed, experience of PD-specific wearables]

Others described how mobility in particular could change considerably throughout the day, meaning even a well-intentioned clinical observation gave an incomplete and unrepresentative picture:

*...when I go to see a consultant and he watches me walking, that snapshot is not at all indicative of what my daily walking looks like...you’re conscious of being watched and you walk differently as a result of that. As I say, my walking changes so much throughout the day that, yeah, I think [wearable devices] would be a great idea.*

[P75, female, 63 years, 9 years diagnosed, experience of PD-specific wearables]

Carers shared these concerns, describing clinical assessments as subjective and time-limited snapshots that depended on how the person presented during the appointment and what they could recall or communicate, making it difficult to convey the full picture of their condition to clinicians. As one carer reflected:

*...basically, you see a neurologist once every six months...If my husband’s feeling good when he goes... the doctor’s not seeing him at his worst...So, it would be good if there was something that the doctor could maybe just...download, have a quick look and say, “Yes, okay, you’re good today, but last night you were really bad. Maybe we can look at the hours that are bad. Maybe we can adapt the medication to that.”*

[PC13, female, 60 years, supporting someone diagnosed for 17 years, no wearable device experience]

Wearables, in contrast, were seen as offering something that clinic-based assessments could not: a continuous objective record of everyday functioning that went beyond what any single clinical encounter could capture:

*That’s really important. The objectivity of getting correct information rather than the subjectivity of how she’s feeling today... is really important...Also, when a neurologist is examining a patient... it’s only a snapshot in time. The device is not just a snapshot in time, it’s a continuous stream of data coming in…*

[PC8, male, 78 years, supporting someone diagnosed for 13 years, no wearable device experience]

In several accounts from rural participants, the benefit of monitoring was linked to long periods between appointments and limited access to local PD services. For these participants, remote monitoring was viewed as a potential means of maintaining clinical oversight between appointments.

Wearable devices were also valued for reducing reliance on patient recall and subjective symptom descriptions, which were described as difficult and often unreliable, especially given the infrequency of appointments. People with PD noted that describing symptoms requires interpretation, which can vary between individuals, whereas objective measurements could provide a shared and more consistent understanding:

*It’s often really difficult to describe symptoms, isn't it? If you can measure things instead of describing them, you know, my description of stiffness or freezing is going to be different to someone else’s description, and it’s going to be based on your personal experience. Whereas if there’s something you can actually measure, it does feel like a much better way of doing it.*

[P4, male, 50 years, 1.5 years diagnosed, experience of PD-specific wearables]

Perceived usefulness, however, depended on whether participants expected monitoring to reveal changes that could inform care. Some carers felt that monitoring would be most valuable earlier in the condition, when mobility might change more rapidly and device use could become easily embedded within established routine. Others questioned its usefulness when symptoms were relatively stable or when the person they supported was perceived to be at a more advanced stage, where the potential for intervention was considered less productive:

*I can see a huge benefit in that in the early stages of Parkinson’s in terms of people’s mobility changing...She would be able to monitor her mobility, but from my observation, it isn’t changing greatly at the moment.*

[PC3, male, 81 years, supporting someone diagnosed for 5 years, experience of consumer wearables]

#### Data as Personal Evidence

People with PD anticipated that wearable data could be a tool for self-advocacy and additional validation, particularly in situations where their experience was difficult to articulate or sometimes dismissed. One participant described how the data could support discussions about symptom patterns that were not typical or widely known:

*It would be really important, I think, to track those data. So, I’d go to my doctor and say, “Hey, listen, during my period or two days before my period, my meds don’t work at all. Here’s the data to show.” He believes me, but other doctors I know are very dismissive.*

[P14, female, 45 years, 3 years diagnosed, experience of PD-specific wearables]

Others felt that having objective data would alleviate the pressure on their memory and provide a more reliable basis for clinical decision-making:

*...as long as I was able to present it to someone that could understand it, the data, and could therefore make decisions upon my health and medicine and so on and so on, my treatment, based upon factual data, as opposed to just me trying to remember…*

[P17, male, 64 years, 4 years diagnosed, experience of consumer wearables]

One participant described how the data could be used to evidence the need for changes in treatment:

*I think it would be [helpful], because if you went to your consultant, you could say, “I think I probably need to go on four now a day, and I can prove that because of this [the data].”*

[P62, female, 65 years, 1 year diagnosed, experience of consumer wearables]

Carers similarly viewed wearable data as a means of substantiating concerns that were otherwise difficult to demonstrate in routine appointments. One carer described how data collected while her husband wore a monitoring device for a research study had supported her concern that his prescribed medication dosage was insufficient:

*We all keep saying he’s under-medicated. [wearable device] showed it.*

[PC5, female, 62 years, supporting someone diagnosed for 17 years, experience of PD-specific wearables]

The view that data could be used as personal evidence also created expectations of ownership and control. Participants, particularly people with PD, wanted access to their own data and the ability to use it in decisions about their care, as one participant expressed in terms of clear personal ownership:

*It’s not the clinician’s data, it’s my data.*

[P75, female, 63 years, 9 years diagnosed, experience of PD-specific wearables]

Others highlighted the importance of data being accessible to the wearer, rather than being transferred to the clinician without feedback to the individual:

*I think it would be good if the person, you know, who was wearing the device, could actually see the data as well...rather than it just being siphoned over. So, if there was some user-friendly data that could be seen by the patients, I think that would be useful.*

[P65, female, 57 years, 3 years diagnosed, no wearable device experience]

Although many participants expressed little concern over privacy, they often acknowledged that these issues could matter to others and emphasized the importance of clarity about what data would be collected, where it would go, and how it would be used. Privacy was often considered in relation to perceived benefit, with many willing to accept a degree of data sharing in exchange for potential clinical value:

*I suppose privacy is one but, at the end of the day, the world is full of information gathering devices, half of which we don’t even know are monitoring us...I’m ready to trade a bit of privacy for more time having fun.*

[P1, male, 70 years, 11 years diagnosed, no wearable device experience]

However, access to data alone did not provide agency. Across both groups, participants emphasized that wearable data could only be useful as evidence if outputs were presented in a clear and meaningful way so that they could be acted on between appointments. One participant identified this as a key limitation of previous wearable use:

*...it has to be manageable, I have to be able to understand it...That’s part of the problem I have with the [wearable device] sometimes is just trying to understand what it’s trying to do...*

[P2, male, 60 years, 2 years diagnosed, experience of PD-specific wearables]

Furthermore, some participants noted that having access to a device and previous experience of wearable technology did not guarantee that its outputs were understandable or actionable. Difficulties may extend beyond accessing and operating a device to interpreting and acting on outputs, even where digital access and basic operational skills are present. One older participant, already using a consumer device that reported walking speed, symmetry, and steadiness, valued the device but could not understand what its results meant:

*I think [wearable device] is good, but I don’t know what these results mean.*

[P61, female, 81 years, 7 years diagnosed, experience of consumer wearables]

#### Complementing, Not Replacing, Care

Both groups felt that wearable data could improve the efficiency and usefulness of clinical time. People with PD expressed frustration with short consultations and long waits between appointments, which left them feeling that important information could be missed. They anticipated that if clinicians could review the data before an appointment, consultations could focus more directly on care and treatment decisions:

*I think [wearable devices] would be an excellent idea...you have this very short time in with the consultant...[the clinician] could be a lot more informed before you got there. So, your 20 minutes, 15 minutes or whatever, is much more used with important things rather than you describing it, it could be like, “Well, what can we do to help?”*

[P62, female, 65 years, 1 year diagnosed, experience of consumer wearables]

Some carers envisaged that wearable data could support proactive follow-up and more efficient allocation of specialist time, directing attention toward people whose mobility had changed rather than relying on a fixed schedule:

*...if you’ve got data... you can get that data and say, “I don’t actually need to see [person with PD] because he’s actually stable...”... You can have it linked to the system so it will just go, “It’s time you saw [person with PD]. He’s really changed in the last three months.”*

[PC22, female, 73 years, supporting someone diagnosed for 6 years, experience of consumer wearables]

Underlying all these views, however, was a consistent expectation that wearables would not replace existing clinical contact. Some participants expressed concern that remote monitoring could be used to justify reducing personal contact and stressed that acceptability depended on the assurance that wearables would complement, rather than substitute, existing care:

*The only concern would be, “Was it trying to short-circuit the personal contact?”*

[P91, male, 66 years, 6.5 years diagnosed, experience of consumer wearables]

### Making Technology Fit the Person

#### Overview

Acceptability depended on whether wearable devices could be integrated into everyday life without adding practical or emotional burden. Across both groups, participants preferred devices that were lightweight, comfortable, and discreet, with minimal maintenance and adequate battery life. These preferences were shaped by the fluctuating nature of PD, the implications of wearing a visible device, and the potential emotional impact that comes with continuous monitoring.

#### Usability Defined by the Difficult Moments

Participants judged usability by whether a device would remain workable as symptoms fluctuated, particularly during *“off”* periods when motor symptoms were more pronounced. Putting on or taking off a wrist-worn device might be manageable sometimes but become difficult when tremor increased or dexterity reduced. These difficulties underpinned a preference for passive monitoring that required little to no interaction and remained functional when symptoms were most pronounced. Participants therefore required simple devices with large controls and easy-to-navigate interfaces that avoided fiddly features requiring fine motor control, such as small charging ports or intricate fastenings:

*I guess ease of use is a problem...when I’m off I wouldn’t be able to go through menus and things a lot. It would have to be really simple: large buttons, easy access*

[P57, male, 42 years, 10.5 years diagnosed, no wearable device experience]

Usability also depended on participants’ wider health needs and personal circumstances. Adhesive patches could irritate sensitive skin, some body locations were difficult to reach, and any audible or vibrating feedback could become intrusive. Existing equipment, such as back supports, could also make a lower-back sensor impractical. Therefore, an acceptable device would need to be workable during periods of more severe symptoms and accommodate individual health needs.

#### Managing Visibility and Discretion

Participants’ acceptance was also influenced by the device’s appearance and how it might be interpreted by others. Bulky or highly visible devices were considered obtrusive and less desirable for long-term use, especially in public settings. Those resembling familiar accessories, such as watches, could blend into everyday life, whereas medical-looking or unfamiliar alternatives risked attracting attention or inviting unwanted assumptions. One participant anticipated feeling self-conscious about more noticeable devices depending on where they were worn:

*I can imagine that you'd feel a bit awkward in public, and particularly if you’re wearing them on your ankles, because they look like tags that you’re under curfew or something.*

[P56, female, 45 years, 3 years diagnosed, experience of PD-specific wearables]

For some participants, discretion was also connected to retaining control over whether their condition became visible to others. This was especially important for younger participants managing how and when their condition became known in social situations, where a device could draw attention to something they had not yet chosen to disclose. One participant with young-onset PD expressed concerns that a noticeable device could invite pity and only considered it acceptable if it remained small and unobtrusive as not to draw attention:

*I wouldn’t want, like, people to see it on me...I guess I don’t want people to feel pity...I’d be alright with that, as long as it’s a small device.*

[P87, male, 53 years, 7 years diagnosed, experience of consumer wearables]

#### The Emotional Weight of Monitoring

The prospect of continuous monitoring carried an emotional weight for some participants. By making symptom patterns and changes over time visible, wearable data could either provide reassurance or intensify awareness of disease progression. For some, data showing that their condition remained relatively stable could reduce uncertainty and provide reassurance:

*I think it’s a bit of reassurance...having a thing that says...“You're no worse than you were six months ago,” is a lot more comforting than this constant, “Ooh, what’s that? What’s that twitch? Is that something that’s part of it?”*

[P4, male, 50 years, 1.5 years diagnosed, experience of PD-specific wearables]

At the same time, some participants anticipated that continuous monitoring could be emotionally challenging because it might draw attention to their symptoms and make deterioration feel more visible. A minority expressed a reluctance to wear a device because they feared that monitoring would heighten negative awareness of their condition:

*I am scared. Seeing and thinking about the symptoms in my head...[with a wearable device] I will see, understand and see that I am becoming worse day by day.*

[P203, female, 61 years, no wearable device experience]

### Reassurance, Responsibility, and the Burden of Care

#### Overview

For carers, the value of monitoring came from its potential to provide reassurance and alleviate some of the mental burden of caring. However, this value depended on the information being reliable and on monitoring not transferring additional practical responsibilities to them.

#### Relief From Constant Vigilance

Carers frequently spoke about the constant vigilance involved in supporting someone with PD whose mobility was unpredictable and the anxiety associated with leaving them alone, even briefly, in case of a fall or sudden deterioration in movement. Falls were reported as a central concern and a marker of declining mobility, and wearables were anticipated as a way of extending oversight beyond the carer’s immediate presence. Fall detection and alerting systems were described as the most tangible and meaningful benefit of remote continuous monitoring, offering a safety net that could reduce the mental load of caring and provide confidence that they would be alerted if something happened, supporting greater independence for both the carer and the person with PD:

*I can see the benefit of [wearable devices] being a useful support...when I first started to leave [person with PD] on her own...I’d go for a walk, and obviously it was on my mind all the time. So, if there was a signalling facility on it, you know, for help.*

[PC21, male, 88 years, supporting someone diagnosed for 20 years, experience of consumer wearables]

Access to wearable devices was therefore seen as a way of alleviating the anxiety that carers described as part of their everyday role, helping them feel better supported in ensuring the person with PD was safe:

*...I want to make sure he’s okay and if he needs anything either that I can provide or I need to negotiate for him to be provided with...to have that information would be really reassuring.*

[PC4, female, 53 years, supporting someone diagnosed for 2 years, no wearable device experience]

Carers also raised concerns that the person with PD might underreport or downplay difficulties and viewed wearables as a further source of reassurance, providing objective awareness of symptoms that might otherwise go unacknowledged:

*...my concern is that he might downplay things. So, yes, it would definitely resolve that anxiety.*

[PC4, female, 53 years, supporting someone diagnosed for 2 years, no wearable device experience]

#### Supporting Care Without Adding to It

For carers, the sense of reassurance offered by wearables was conditional. First, acceptability depended on alerting systems feeling reliable, neither triggering false alarms unnecessarily nor failing to detect a real event, as oversensitive devices were expected to increase, rather than alleviate, anxiety:

*...as long as it worked and was effective...If it was so sensitive that it would go off at a moment’s notice, I’d be more anxious.*

[PC14, female, 76 years, supporting someone diagnosed for 19 years, experience of PD-specific wearables]

Alert fatigue was a related practical concern. While many carers valued safety alerts, some stressed the need for balance and expressed a preference for trended summaries rather than constant notifications:

*...what I don’t want is I don’t want an alert every 10 minutes for a different thing from them... So, it’s how you get that balance of what do you need to know?... if you’ve got a platform that you can see all those trended data, then, yeah, I can go into it and have a look, as long as they’re happy to do that.*

[PC7, male, 56 years, supporting someone diagnosed for 7 years, no wearable device experience]

Carers also anticipated that wearable devices could introduce a new layer of responsibility into their caring role. Where the person with PD experienced difficulties with technology, memory, or routine, carers anticipated that the practical demands of managing devices, such as charging, troubleshooting, and ensuring the device was recording correctly, would fall to them. Some expressed concern that wearables could become *“another element”* of caring:

*You know what I mean, is it just going to add another element of things... I wouldn’t want to end up doing it if it ended up onerous for me... I don’t want anything that creates extra work for me.*

[PC5, female, 62 years, supporting someone diagnosed for 17 years, experience of PD-specific wearables]

Others described this shift in burden more openly, expecting that interaction demands, such as inputting data and ensuring processes were followed correctly, would fall to them rather than the person with PD:

*...it’s the fact of inputting the data, or doing things, that would stress him out... because it’d stress me out to make sure it was done properly. So, it’s the carer...*

[PC12, female, 62 years, supporting someone diagnosed for 2 years, experience of consumer wearables]

This explains why passive monitoring was frequently preferred. Carers noted that the person with PD would struggle with smartphone-based tools or anything that required remembering steps and that memory made it difficult to rely on them to engage consistently with technology. The device would therefore need to operate entirely in the background, without active input:

*Anything that involves [person with PD] and the smartphone is no... But it’d have to be something that just operates without [person with PD] having to do anything.*

[PC23, female, 73 years, supporting someone diagnosed for 16 years, no wearable device experience]

Preferences for passive monitoring were not simply convenience; it could reduce the mental load of caring only if it did not add to the practical one, and a device requiring active management would transfer work to a person it was intended to support.

More broadly, a few carers highlighted the importance of being recognized as stakeholders in both the research and implementation of wearable devices. These concerns about visibility and recognition reflected a wider feeling that the impact of PD on carers was frequently overlooked, despite the condition shaping the lives of both the person with PD and those who supported them:

*We have this uninvited guest in our life who is not going to go away... that’s going to affect your lives, both of your lives. And I think both sides of that, the sufferer and the supporters, need to have something...It’s easy to forget the carers, the supporters.*

[PC2, male, 70 years, supporting someone diagnosed for 17 years, experience of PD-specific wearables]

Concerns about recognizing those expected to support wearable monitoring extended to the wider care system. Carers also questioned how this type of monitoring would be managed in formal care settings, where staff capacity and digital literacy could vary. They emphasized that both the device and its outputs would need to be straightforward to use and interpret:

*If you think about the care industry, it’s mostly people who are on minimum wage and often will be part-time and filling in. So, how do we make sure that they have a suitable understanding of both the technology but also how to interpret data?...How do we train people?...how you visualise that is really key to make it super simple.*

[PC7, male, 56 years, supporting someone diagnosed for 7 years, no wearable device experience]

## Discussion

### Principal Findings

This qualitative study provides an in-depth exploration of how people with PD and informal carers perceive wearable devices for remote continuous mobility monitoring. While previous qualitative work has examined views of wearables in people with PD [23,27], to our knowledge, this is the first study to examine carer perspectives as a direct analytical focus alongside those of people with PD, and to do so with a diverse sample recruited specifically to include groups at heightened risk of digital exclusion and underrepresentation in PD research. Although digital health research has increasingly acknowledged the potential of wearable technology in PD, the views of informal carers remain much less frequently considered, despite their central role in everyday support [30,32,50]. Across both groups, participants saw clear value in wearables for generating objective real-world evidence of mobility beyond what brief clinical appointments could provide. People with PD described consultations as artificial “snapshots” in which they “performed” for the clinician, often making them appear better than usual, consistent with the Hawthorne effect, where people modify their behavior when aware of being observed [51]. Carers similarly felt that the full picture of day-to-day functioning was rarely conveyed to clinicians and that the person they supported was often seen at a relatively good moment rather than their worst. These accounts echo longstanding concerns that episodic clinical assessment methods fail to capture symptom variability and context-specific difficulties in PD [12,14,16,17,23] and reinforce growing interest in wearables as a way of supporting more precise monitoring of progression and treatment response [9,11,18,21,31].

27

The present findings extend this literature by showing how people with PD positioned wearable data as a tool for reshaping the consultation itself. They described how it could support more productive appointments, better-informed treatment discussions, and self-advocacy. This was partly because wearables were perceived to reduce reliance on memory and subjective description, challenges previously highlighted as barriers in PD care [16,17]. Carers, in contrast, focused on the practical side, hoping wearables would give them confidence to leave the home, provide reassurance about safety and falls, and ease the constant vigilance of caregiving [50,52], while recognizing that managing the device could add to their workload [52,53]. People with PD foregrounded clinical utility and self-management, whereas carers were more concerned with risk management, anticipation of problems, and the practical realities of supporting someone whose mobility may change unpredictably [50]. This contrast reinforces the view that PD mobility is felt as a shared experience within the care relationship and suggests that the value of wearable monitoring may ultimately depend on how well it supports the wider care dyad rather than the person with PD alone [52,54].

Although both groups were broadly open to wearable monitoring, acceptability was conditional, consistent with wider conceptualizations of acceptability as a multidimensional construct shaped by perceived burden, usability, ethical concerns, and effectiveness [24]. People with PD were willing to tolerate some inconvenience, including visibility or loss of privacy, when they believed the device had a clear purpose and would meaningfully inform their care. Carers were more cautious, with acceptability depending less on clinical promise and more on whether the device could fit into daily life without adding to the demands of caregiving. Together, these priorities align with the wider literature on self-management in PD, which emphasizes that digital tools are most likely to be adopted when they fit within existing routines, demand minimal additional effort, and offer a clear and personally relevant benefit to those involved [55-57]. Acceptability was further shaped by emotional responses to monitoring; some people with PD anticipated that wearable data would be reassuring by reducing uncertainty or confirming condition stability, while others felt that continuous monitoring risked heightening symptom awareness and making deterioration feel more visible. These 2 perspectives echo previous work showing that wearables can be both beneficial and emotionally challenging for people with PD [23,27] and reinforce the conceptualization of acceptability by Sekhon et al as encompassing affective as well as practical dimensions [24].

A further concern, raised particularly by carers, was that wearable technology risked adding to the work of those already providing substantial support, especially where the person with PD experienced cognitive difficulties or low confidence with technology. This resonates with wider discussions in digital health and long-term condition management about the hidden labor involved in sustaining home-based monitoring, and the risk that technologies framed as empowering may, in practice, redistribute responsibility onto patients and carers rather than relieve it [52,53]. Tools intended to support autonomy could simultaneously generate new forms of dependence on informal carers, particularly in progressive conditions where the capacity to engage with technology may decline over time. This explains why passive monitoring was preferred, as a way of ensuring that the device did not become another task within an already demanding role. Carers should therefore be recognized as key stakeholders in the design and implementation of wearable monitoring because its success may depend partly on whether it lessens or exacerbates the burden of caregiving [52-54].

Esthetics and functionality were also central to acceptability across both groups, with participants favoring designs that were unobtrusive, familiar, and easy to incorporate into daily life. Watch-like devices were generally preferred over more visible or medical-looking alternatives, reflecting concerns about stigma and self-consciousness in public. This finding is consistent with wider work showing that how a device is perceived socially, not just how well it performs, shapes whether people are willing to wear it long term [23,27]. However, these preferences may exist in tension with clinical utility, as wrist-worn devices may capture less detailed or accurate information about gait and mobility than lower-back sensors or multisite configurations [46,47]. Compounding these general preferences were condition-specific challenges; tremor, reduced dexterity, and symptom fluctuations could turn seemingly simple tasks, such as fastening straps, navigating menus, or managing charging, into significant obstacles, echoing previous concerns that PD symptoms create distinct demands for digital engagement [23,25]. These findings underline the importance of condition-sensitive, co-designed wearable design for PD.

A key implication of this study is that acceptability depended on remote continuous monitoring complementing rather than replacing clinical care. Although both groups valued the possibility of more informed and efficient consultations, some worried that wearable monitoring could be used to justify reducing in-person appointments altogether. This concern is particularly relevant in the context of England’s 10 Year Health Plan and its proposed shift from “analog to digital” [58], with participants’ accounts suggesting that they are open to digital tools when they enhance care, but not when they are used to substitute the human contact that remains central to managing a complex progressive condition. This was especially evident among several rural participants, who described limited access to specialist services and valued continuous monitoring as a means of maintaining clinical oversight across long intervals between appointments. These participants may benefit substantially from remote monitoring but could also feel the greatest loss if its introduction led to further reductions in specialist contact. Clinically, this points to wearable monitoring being most acceptable as an adjunct to, rather than a replacement for, routine assessment in PD, particularly where it can capture symptom fluctuations, inform medication management, and improve the quality of short and infrequent appointments [9-11,21,23]. Whether these benefits could be realized in practice remains uncertain; it would depend on appropriate clinical pathways, staffing, data infrastructure, and clinical engagement. The present findings capture participants’ expectations rather than evidence that these benefits would be achievable in practice. Implementation should therefore ensure real-world mobility data are only collected where a clear care pathway exists for reviewing and acting on them and with outputs designed to be interpretable not only to clinicians but also to people with PD and carers themselves.

The need for interpretable outputs highlights an important distinction: participants valued wearables for producing “objective” data, but objective measurements are not the same as clinically meaningful evidence. Wearables can measure aspects of mobility, such as walking speed, stride length, turn duration, and gait variability, which may help identify medication effects, fall risk, or changes over time [59,60]. However, before these measurements can guide care, they must be validated for their intended purpose and interpreted within the relevant clinical context [61]. Interpretability is therefore as important for clinicians as it is for people with PD and carers because clinicians need to know whether a change is important and what action it should lead to [62]. Clinicians have similarly identified a need for concise summaries of relevant measures, presented as trends with flags for important changes, and standardized actions to implement, rather than raw sensor data [62]. These conditions, alongside the limited availability of established care pathways and clinicians’ own concerns about clinical utility and workflow [63-65], help explain why wearable monitoring in PD remains largely confined to research settings. However, progress toward implementation is evident, with calls to integrate remote monitoring into PD care [66,67] and early examples of these approaches being introduced into routine care [68,69].

At a policy level, these findings speak to the growing enthusiasm for digital technology in long-term condition management, which often anticipates that technology will improve monitoring, personalize care, and enhance service efficiency [9-11,18,32]. This study suggests that these benefits are not guaranteed and may come at a cost if implementation overlooks usability and the practical burdens of adopting new technologies [54]. Policy efforts to develop and implement wearable devices in PD should therefore be accompanied by clear standards for accessibility, co-design, transparent data governance, and clinical integration. Transparency is particularly important, given that participants valued clarity about what data would be collected, who would have access to it, and how it would be used, reinforcing the importance of trust in sustained engagement with digital technologies. Digital health policy should also move beyond a patient-only framing of technology adoption because informal carers are often essential to the successful use of digital health tools in PD yet remain underrecognized in both research and service planning [50,52,53]. A further gap concerns formal carers, who are likely to play a growing role in supporting wearable device use within residential, nursing, and home care settings, yet whose perspectives remain largely absent from the existing literature despite evidence that formal social care forms part of the wider PD support network [70]. Addressing this gap will be essential if wearable monitoring is to be integrated into PD care in ways that are feasible, clinically meaningful, and acceptable across the full support network.

Several limitations should be considered. This study explored anticipated acceptability of wearable devices rather than experiences of using these technologies in routine care. Although some participants drew on prior experience of PD specific (people with PD: 28/72, 39%; carers: 4/20, 20%) or consumer wearables (people with PD: 16/72, 22%; carers: 6/20, 30%), many had no prior experience (people with PD: 28/72, 39%; carers: 10/20, 50%), and a substantial proportion were responding to a proposed technology rather than lived experience. The findings therefore capture expectations, preferences, and concerns, but not how acceptability evolves through prolonged use. Relatedly, participants’ previous experience is likely to have shaped how they interpreted the device descriptions. Those with experience, especially with PD-specific research devices, could draw on first-hand examples when discussing usability, whereas others evaluated a more hypothetical concept. Differences in digital literacy and familiarity may also have influenced how readily they could envisage a device’s role in everyday care. A further limitation concerns the interview guide; before exploring preferences, participants were told that lower-back sensors generally offer greater measurement accuracy than wrist-worn devices. This information was intended to support informed responses regarding technologies with which participants often had limited experience; however, it may have shaped participants’ responses, including their preferences for device placement. Nonetheless, participants frequently preferred wrist-worn devices despite being informed of the greater accuracy of lower-back sensors and readily expressed concerns about the technology, suggesting responses were not solely directed by this framing.

The purposeful recruitment of participants often excluded from PD research was a key strength, particularly given concerns about lack of diversity in the field [29]; 65 (71%) of 92 participants had at least one characteristic associated with underrepresentation in PD or digital health research. Our aim was not to explore the experiences of separate underserved groups but to ensure that people typically absent from this research and at heightened risk of digital exclusion [28] contributed directly to a shared understanding of acceptability. Understanding the specific needs of individual groups remains important but would require studies with sufficient representation. Nonetheless, the sample remained predominantly White and relatively highly educated, and the most socioeconomically deprived and ethnic minority communities remained underrepresented. Reaching these groups fully is likely to require dedicated and adequately resourced recruitment through trusted community organizations, and we recommend this as a priority for future work, consistent with wider calls to strengthen diversity and inclusion in PD and care [29]. This community-based recruitment approach meant that PD diagnosis was self-reported, with no formal clinical assessment of disease stage or severity, and those who volunteered may have been more open to health technologies than those who chose not to take part, so the findings may somewhat overrepresent openness to wearable monitoring, although PPIE throughout the study supported the relevance and grounding of the findings.

Analyzing a large dataset using RTA, which understands rigor as sustained interpretative engagement [71], presents a recognized challenge. Interpretative depth was supported by the lead analyst conducting 43 of the 92 interviews and repeatedly reading all transcripts, alongside reflexive and postinterview notes that documented both similar and divergent accounts. A sole analyst led coding and theme development throughout, while regular team discussion provided opportunities to develop and critically reflect on emerging interpretations.

### Conclusions

This study shows that wearable devices for remote continuous mobility monitoring were generally acceptable to people with PD and informal carers, particularly when they were perceived to capture mobility more accurately than clinic-based assessment and to support safer, more informed, and proactive care. However, acceptability was conditional, depending on devices being easy to use, interpretable, and integrated into care in ways that supported rather than replaced clinical relationships. The findings also highlight the central role of carers, whose needs, burden, and perspectives shape whether these technologies are workable in practice. Overall, the successful implementation of wearable mobility monitoring in PD will require more than technical validity. It will depend on person-centered and carer-aware design, and on thoughtful clinical integration capable of turning data into meaningful support.

## Supplementary material

10.2196/98987 Multimedia Appendix 1 Interview topic guide for people with Parkinson disease.

10.2196/98987 Multimedia Appendix 2 Interview topic guide for carers.

## Abbreviations

CLEARS: conditions, education, age, residence, and socioeconomic status
EPSRC: Engineering and Physical Sciences Research Council
EU IMI: European Union Innovative Medicines Initiative
NHS: National Health Service
NIHR: National Institute for Health and Care Research
PD: Parkinson disease
PPIE: patient and public involvement and engagement
RTA: reflexive thematic analysis
