# Supplementary material for: Acceptability of Wearable Digital Devices for Remote Continuous Mobility Monitoring in People With Parkinson Disease and Carers: Qualitative Study
Source: J Med Internet Res. 2026 Oct 30;28:e98987. doi: 10.2196/98987 (PMC13722101; doi:10.2196/98987)
Supplement: Supplementary file 1 [file jmir-v28-e98987-s001.docx]

**Interview Topic Guide**

*Please note the interview topic guide is a living document. The design is iterative to tailor the interview to the needs of the participant, and the issues that they feel are most important. As analysis occurs concurrently, the topic guide is likely to be adapted over time - questions might be added, amended or omitted – and used flexibly.*

**Guide overview**

| **Topic** | **Overview** |
| --- | --- |
| 1. Background information | - Consent - Background information about participant and their Parkinson’s |
| 2. Symptom experiences and impact | - Identifying symptoms experienced by the participant and impact on daily life |
| 3. Mobility and digital mobility assessment | - Identifying mobility-specific experiences and impact on daily life - Participants acceptability of digital mobility assessments |
| 4. Quantifying symptoms and impact | - 0-10 scale to measure bothersomeness of symptoms |

**Introductions**

First, I’d like to thank you for joining this interview and participating in our study.

To remind you, the purpose of this interview is to explore your experience with Parkinson’s including your symptoms and how they affect your daily life. We will also ask specifically about your mobility (i.e., walking) and explore your views on measures to monitor your Parkinson’s, such as wearable devices.

With your permission, I will audio-record our conversation today so that I can refer back to it later on to ensure we accurately record your experience. This interview is confidential, you will not be identifiable in any of the reports that result from these interviews, and you can stop at any time or take breaks if you wish.

Finally, I will be taking a few notes during our conversation.

- Before we begin, do you have any questions about the study or consent information?
- Before I start recording, I would like to ask a couple demographic questions so we can understand our participants a bit more – is that okay?
- May we audio-record the interview? **[BEGIN RECORDING]**
- Can you reconfirm that you have agreed to be recorded and have consented to participate in this study?

**Section 1: Background information**

*Note: this section aims to gather contextual information and to serve as ‘icebreakers’ for further discussion*

1. Could you tell me about yourself and your life with Parkinson’s?

**Prompts:** when were you diagnosed; how long do you think you had Parkinson’s for before you were diagnosed; why do you think that; what did you notice; were there changes in what you were able to do/how you felt?

Do you take any medication and if so, how frequently do you take it; have your symptoms changed since being diagnosed?

2. How has being diagnosed with Parkinson’s impacted you?

**Prompt:** How has your Parkinson’s changed overtime?

**Section 2: Symptom experiences and impact**

I’d like to focus on your symptoms and the affect they have on your daily life, e.g., daily activities, work, leisure, social life, emotional impact, etc.

1. On a typical day, what symptoms do you experience because of your Parkinson’s?

*Note: run through the following questions for each symptom*

a. Can you tell me about your experience of *[symptom]*
b. How does this affect your daily life?

Prompt: how much does it bother you? Is there anything you are not able to do because of this?

c. How do you manage this?

Prompt: are there any treatments/therapies that help with this?

d. How often does this happen?

I will now take you through a list of symptoms that other people with Parkinson’s have reported experiencing. Your experience may be different.

*Note: run through list of symptoms asking the participant if they have experience them. Ask the following questions again for each symptom they mention experiencing.*

e. Can you tell me about *[symptom]* and how it affects your life?

**Prompt:** is there anything you are not able to do because of this symptom?

f. How often do you experience this symptom?
g. How do you manage this symptom?

2. Are there any other symptoms that you experience because of Parkinson’s that I have not addressed?

****Check if they are happy to continue or need a break****

**Section 3: Mobility and digital mobility assessments**

Thank you for telling us about your experience with Parkinson’s. The work that we are doing focuses on mobility in Parkinson’s (i.e., your ability to move and walk freely) and the impact that has on your daily life. So, now I will ask you a few questions about your mobility.

1. Specifically thinking about mobility, could you talk me through a typical day and the activities you usually perform?
2. Are there any activities that you find difficult or avoid due to limited mobility?

**Prompt:** getting out of bed, showering, walking from room to room, household cleaning, social activities, shopping, exercise

3. How do you typically cope with these difficult activities, do you have any assistance or aids helping you out?

With our work we are also wanting to explore your views on the use of wearable devices to measure your mobility (e.g., a monitor or a watch, placed on your lower back or wrist). A monitor worn on the lower back may provide more accurate information about walking and movement than a wrist-worn device.

Monitoring your mobility through these devices could be used by clinicians in making decisions about how to understand and treat your Parkinson’s.

3. What do you think about wearing a device that could help monitor your Parkinson’s?

**Prompt:** where would you prefer the device to be placed on your body (e.g., wrist, lower back); why is that?

4. What do you think could be good about these devices?

**Prompt:** what would you like to get out of these devices; what would you like them to capture?

5. Do you have any concerns about these devices?

**Prompt:** comfort, ease of use, data privacy etc.

6. Would you be willing to wear this device to monitor your Parkinson’s?

IF YES, how long would you be willing to wear this device? – e.g., a day, a week, a month, etc; how often would you be willing to wear this device? – e.g., all day, a few hours, etc.

**Section 4: Quantifying symptoms and impact**

I am now going to go through a few questions, they may feel repetitive, but they make sure we can understand which of your symptoms bother you the most.

So, for the symptoms you mentioned experiencing, I would like to quantify how much they affect your daily life. We will use a scale of 0-10, where 0 means that the symptom does not disturb your life at all, 5 means the symptoms moderately disturbs your life, and a 10 means that the symptom is extremely disturbing.

*Note: run through the following 2 questions for each symptom*

1. How much does [symptom] disturb your life on a typical day using the 0-10 scale?
2. Have there been times in the past month when this symptom was worse than on a typical day?

*Note: ask the following question after the list of symptoms is complete*

3. From all the symptoms that you experience, which symptom bothers you the most?
   a. Why does this symptom bother you the most?
4. From all the symptoms that you experience, which symptom would you most like to be improved?

**Conclusions**

- Before we end the interview, do you have any other thoughts that you would like to share?
- Thank for participation and switch off recording.

**Symptom list**

| **Symptoms** | **Yes/No** |
| --- | --- |
| Tremors |  |
| Slow movement |  |
| Muscle stiffness (rigidity) |  |
| Muscle cramps |  |
| Balance issues |  |
| Fatigue, tiredness, weakness |  |
| Freezing movements |  |
| Falling |  |
| Pain in your neck, chest, back, arms, legs or feet |  |
| Shuffling walk (festination) |  |
| Jolting movements |  |
|  |  |
|  |  |
|  |  |
