# Supplementary material for: Acceptability of Wearable Digital Devices for Remote Continuous Mobility Monitoring in People With Parkinson Disease and Carers: Qualitative Study
Source: J Med Internet Res. 2026 Oct 30;28:e98987. doi: 10.2196/98987 (PMC13722101; doi:10.2196/98987)
Supplement: Supplementary file 2 [file jmir-v28-e98987-s002.docx]

**Interview Topic Guide**

*Please note the interview topic guide is a living document. The design is iterative to tailor the interview to the needs of the participant, and the issues that they feel are most important. As analysis occurs concurrently, the topic guide is likely to be adapted over time - questions might be added, amended or omitted – and used flexibly.*

**Introduction**

First, I’d like to thank you for joining this interview and participating in our study. To remind you, the purpose of this interview is to explore your experiences of supporting someone with Parkinson’s disease, in particular mobility-related challenges and how they affect daily life. We will also explore your opinions on the use of technology to assist in that care.

With your permission, I will audio-record our conversation today so that I can refer back to it later to ensure we accurately record your experience. This interview is confidential, you will not be identifiable in any of the reports that result from these interviews, and you can stop at any time or take breaks if you wish.

Finally, I will be taking a few notes during our conversation.

- Before we begin, do you have any questions about the study or consent information?
- Before I start recording, I would like to ask a couple of demographic questions so we can understand our participants a bit more – is that okay?
- May we audio-record the interview? **[BEGIN RECORDING]**
- Can you reconfirm that you have agreed to be recorded and have consented to participate in this study?

**Section 1: Background Information**

*Note: this section aims to gather contextual information and to serve as ‘icebreakers’ for further discussion*

1. Could you tell me a bit about yourself and the person with Parkinson’s you support?

**Prompts:** your relationship to the person, when they were diagnosed with Parkinson’s and how you have supported them during this time, their condition, do you see yourself as a carer?

2. How did you come to start caring for someone with Parkinson’s?
3. Do they have issues with their mobility? If so, can you expand on this?
   a. How do these mobility issues affect you?

**Section 2: Caregiving Experiences and Challenges**

Now, I’d like to focus on your experience with supporting someone with Parkinson’s.

Prompts to relate to mobility, how the PwPD mobility impact their life. Talk about mobility symptoms, and how it affects the carer.

Is the mobility aspect the most challenging part? Or is there another aspect?

1. Can you describe a typical day in your life as a carer?
2. Have you encountered any challenges when supporting someone with Parkinson’s?

**Prompts:** are there any specific tasks or activities that are particularly difficult or impossible; do you use any strategies or tools to help manage these challenges?

3. Are the mobility issues the most challenging part or is there another aspect more challenging?
4. How does supporting someone with Parkinson’s impact you?

**Prompt:** impact on personal time, social interactions, hobbies, and interests, changes in daily routines, work-life balance, emotional and physical impact.

**Section 3: Technology and Digital Mobility Assessments**

Thank you for sharing your experience. With our work we are also wanting to explore your views on the use of wearable devices to measure mobility (e.g., a monitor or a watch, placed on your lower back or wrist). Monitoring mobility through these devices could provide insights into a person’s condition.

As an example: Some people have shared that they keep track of their mobility using technology. John explained how his device helps him track his movement, tremors, and overall activity. He said it sends him reminders to move around if he has been inactive for too long. Audrey mentioned that she uses a wearable device that syncs with her phone. This device records her movement and alerts her to any significant changes in her mobility patterns. The device also sends her husband an alert if she fallen.

1. Do you use technology as part of supporting someone with Parkinson’s?
2. Do you think technology could fit within your life?
3. What do you think about using technology as a method of monitoring mobility of the person you support?
4. How do you think these devices could be helpful?

**Prompt:** potential benefits; are there any specific features that would be useful?

5. Do you have any concerns about using technology to assist your care?

**Prompt:** comfort, ease of use, privacy, reliability.

6. Would having a device that monitors mobility be helpful for you?

**Prompt:** how could it support your caring responsibilities?

**Conclusions**

- Before we end the interview, do you have any other thoughts that you would like to share?
- Thank for participation and switch off recording.
